# Ethanol Extract of Mao Jian Green Tea Attenuates Gastrointestinal Symptoms in a Rat Model of Irritable Bowel Syndrome with Constipation via the 5-hydroxytryptamine Signaling Pathway

**DOI:** 10.3390/foods12051101

**Published:** 2023-03-04

**Authors:** Lei Wu, Liming Gao, Xiang Jin, Zhikang Chen, Xutong Qiao, Xiting Cui, Jianhua Gao, Liwei Zhang

**Affiliations:** 1 Institute of Molecular Science, Shanxi University, Taiyuan 030006, China; 2 Shanxi Key Laboratory of Minor Crops Germplasm Innovation and Molecular Breeding, College of Life Sciences, Shanxi Agricultural University, Taigu, Jinzhong 030801, China

**Keywords:** Mao Jian Green Tea, ethanol extract, gastrointestinal motility, 5-hydroxytryptamine, gut microbiota, flavonoids

## Abstract

In a previous study, we demonstrated that the hydro extract of Mao Jian Green Tea (MJGT) promotes gastrointestinal motility. In this study, the effect of MJGT ethanol extract (MJGT_EE) in treating irritable bowel syndrome with constipation (IBS-C) in a rat model constructed via maternal separation combined with an ice water stimulation was investigated. First, a successful model construction was confirmed through the determination of the fecal water content (FWC) and the smallest colorectal distension (CRD) volume. Then, the overall regulatory effects of MJGT_EE on the gastrointestinal tract were preliminarily evaluated through gastric emptying and small intestinal propulsion tests. Our findings indicated that MJGT_EE significantly increased FWC (*p* < 0.01) and the smallest CRD volume (*p* < 0.05) and promoted gastric emptying and small intestinal propulsion (*p* < 0.01). Furthermore, mechanistically, MJGT_EE reduced intestinal sensitivity by regulating the expression of proteins related to the serotonin (5-hydroxytryptamine; 5-HT) pathway. More specifically, it decreased tryptophan hydroxylase (TPH) expression (*p* < 0.05) and increased serotonin transporter (SERT) expression (*p* < 0.05), thereby decreasing 5-HT secretion (*p* < 0.01), activating the calmodulin (CaM)/myosin light chain kinase (MLCK) pathway, and increasing 5-HT_4_ receptor (5-HT_4_R) expression (*p* < 0.05). Moreover, MJGT_EE enhanced the diversity of gut microbiota, increased the proportion of beneficial bacteria, and regulated the number of 5-HT-related bacteria. Flavonoids may play the role of being active ingredients in MJGT_EE. These findings suggest that MJGT_EE could serve as a potential therapeutic pathway for IBS-C.

## 1. Introduction

Irritable bowel syndrome (IBS) is a common gastrointestinal disease associated with changes in gastrointestinal motility, secretion, and visceral sensation. It manifests clinically mainly as abdominal pain accompanied by intermittent or persistent irregular bowel movements, as well as abnormalities in stool texture and shape [1,2,3]. Several basic and clinical studies have investigated the etiology of IBS from different perspectives, including the effects of genetic factors, low-grade mucosal inflammation and immune activation following severe gastrointestinal infection, increased intestinal mucosal permeability, changes in gut microbiota, abnormalities in bile salt metabolism, allergies to certain dietary components, abnormalities in neurotransmitter pathways, and changes in brain function [4,5,6,7,8]. Although the pathogenesis of IBS has not yet been fully elucidated, researchers speculate that hypersensitivity and alteration in visceral perception as well as gastrointestinal dysmotility form the main pathophysiological basis of the disorder [9]. IBS can be divided into four subtypes according to the Rome IV criteria: IBS with constipation (IBS-C), IBS with predominant diarrhea (IBS-D), IBS with mixed bowel habits (IBS-M), and IBS unclassified (IBS-U) [10,11]. Approximately one third of all IBS cases are of the IBS-C subtype [12].

Medical treatments for IBS often yield unsatisfactory results, thereby imposing a heavy disease burden on patients [13]. For IBS-D, most clinicians recommend the use of serotonin type 3 receptor (5-HT_3_R) antagonists to block the excessive action of 5-HT on 5HT_3_R and reduce intestinal motility. Common medications used for IBS-C treatment include the serotonin type 4 receptor (5-HT_4_R) agonists, prucalopride and tegaserod [14], the type 2 chloride channel activator, lubiprostone [15], and the guanylate cyclase C agonists, linaclotide [16,17] and plecanatide [18], which can promote intestinal peristalsis. In effect, tegaserod has already been approved for use by the U.S. Food and Drug Administration for the treatment of IBS-C. However, there are age-related limitations and contraindications to the use of lubiprostone and tegaserod, with the latter only being approved for use in a limited IBS-C patient population (women aged < 65 years without cardiovascular disease risk-related contraindications) [19]. Only a few studies have investigated the effects of lubiprostone in Asian patients; therefore, its use in the treatment of IBS-C has not been recommended in South Korea and Japan [20,21]. Given the scarcity of IBS-specific drugs, the treatment of the disease often requires the introduction of other adjuvant strategies, such as good lifestyle habits (eating regular meals and increasing dietary fiber intake), traditional Chinese medications or acupuncture [22,23,24], or acupoint catgut embedding [25] that contribute to improving the condition of the patient.

During history, herbal medicines have also been developed by several countries and regions with specialties to deal with various diseases. Some of them can treat or relieve gastrointestinal disorders. For example, the herbal therapy STW-5 (Iberogast^®^) including angelica roots (*Angelicae radix*), chamomile flowers (*Matricariae flos*), caraway fruit (*Carvi fructus*), St. Mary’s thistle fruit (*Cardui mariae fructus*), balsam leaves (*Melissae folium*), peppermint leaves (*Menthae x piperitae*), greater celandine (*Chelidonii herba*), and licorice root (*Liquiritiae radix*) has been in clinical use in German-speaking countries for decades and is sold as an over-the-counter medicine in Europe. It acts on 5-HT_4_, 5-HT_3_, muscarinic M_3_, and opioid receptors to relieve intestinal spasm and reduce gastric acid secretion [26]. There is much evidence that peppermint oil reduces visceral pain and modulates gastrointestinal motility via TRPM8 and/or TRPA1 receptors [27]. Curcumin, contained in turmeric (*Curcuma longa*), treats abdominal pain as well as other gastrointestinal symptoms present in IBS [28]. *Atractylodes lanceolata* oil was able to ameliorate the rat IBS-D by inhibiting the SCF/c-kit pathway, thereby reducing inflammation and protecting the intestinal barrier from damage via the MLCK/MLC2 pathway [29].

*Dracocephalum rupestre* Hance, called Mao Jian Cao (MJC) in Chinese, is a perennial herb of the *Dracocephalum* genus and the Lamiaceae family that is native to the Northern Shanxi Province of China. MJC is a traditional Chinese medicine, with the effect of relieving headaches, soothing sore throats, subsiding coughs and preventing icterohepatitis [30]. MJC is rich in flavonoids, among which dihydroflavonoids and their corresponding glycosides such as luteolin, luteolin-7-O-β-D glucoside, eriodictyol, and eriodictyol-7-O-β-D glucoside, and terpenoids such as β-sitosterol, betulinol, and betulinic acid, are representative components [31]. Interestingly, the herbal tea made from its leaves as a daily drink to aid digestion is a more popular way. This is because people in these regions often consume the slower-digesting coarse grains. From June ending to September each year, fresh MJC leaves are harvested by locals for making Mao Jian tea (MJT), including MJGT (green tee) or MJBT (black tea). The making method could lead to the production of different metabolites [32]. For example, 130, 136, and 95 compounds were detected in the MJC, MJGT, and MJBT, respectively. There were 28 differential metabolites in MJGT compared to MJC; MJBT had 29. The MJGT-making method led to the significant intensity of some flavonoids such as apigenin, eriodictyol, luteolin, and naringenin, whereas the corresponding glycosides of eriodictyol and luteolin decreased. Interestingly, a similar trend was observed in MJBT, except that the content of some flavonoid glycosides decreased sharply, including the 7-*O*-glucoside of the four flavonoids mentioned above. Common green tea is made from the steamed and dried leaves of the *Camella sinesis* plant. The main active ingredients include tea polyphenols and tea polysaccharides, which have anticancer, antioxidant, neuroprotective, and hypoglycemic pharmacological activities [33]. Green tea contains caffeine (~3%) [34], which enhances the autonomic activity of the vagus nerve, releases acetylcholine, and promotes gastrointestinal motility [35], but such ingredients can also cause euphoria and insomnia after consumption. The caffeine content of MJC is very low at 0.495%, so it does not affect sleep after consumption [36]. In addition, caffeine is one of the main components that form the bitter taste of the tea [37]; therefore, MJC has a lighter taste compared to other common green teas.

Flavonoids have multiple effects on the gastrointestinal tract, including (1) protecting the intestinal epithelium from drug damage and food toxins; (2) regulating the activity of enzymes involved in lipid and carbohydrate absorption; (3) maintaining the intestines of the intestinal barrier; (4) regulating the secretion of intestinal hormones; (5) modulating the gastrointestinal immune system; (6) exerting potential anti-colorectal cancer activity; and (7) shaping the composition of the bacterial flora [38]. For example, quercetin inhibits gastrointestinal toxicity induced by diclofenac and aggravated by ranitidine, improves gastrointestinal bleeding, intestinal permeability, and restores intraluminal pH in rats [39]. The ability of polyphenols in oranges and apples to alter the microbiota of systemic lupus erythematosus (SLE) patients, with their flavonoids increasing the levels of lactobacilli and dihydroflavonols increasing the levels of bifidobacteria, suggests the possibility of correcting the ecological dysbiosis associated with SLE by altering the flavonoid diet [40]. Apigenin showed a dose-dependent relaxation effect on acetylcholine (ACh)-induced muscle strips in the concentration range of 0.1 to 100 μmol/L. Luteolin and quercetin showed a similar performance to apigenin with the exception that, at low doses (0.001–0.1 μmol/L), they were able to further increase the induction effect of ACh [41]. Previously, we demonstrated that MJGT promotes small intestinal propulsion and gastric emptying in normal rats and improves gastrointestinal motility by increasing the abundance of beneficial bacteria [42]. Flavonoids constituted the main active ingredients in MJGT. Considering the easier concentration and higher efficiency for the extraction of flavonoids without the concerns of impacts on toxicity and biodegradability [43], we chose ethanol as the extraction solvent in the present study. A rat model of IBS-C was treated with the ethanolic extract of MJGT (MJGT_EE), and the response of the key enzymes and downstream targets of the serotonin (5-hydroxytryptamine; 5-HT) biosynthesis pathway, which is an essential signaling pathway in the gastrointestinal tract, was investigated.

## 2. Materials and Methods

### 2.1. Sample Collection

MJGT was purchased from Jiufeng Cooperative (Ningwu County, Shanxi Province, China) in December 2018, and samples were conserved at the Chinese Medicine Resources and Sciences Laboratory of Shanxi Agricultural University (JF18001-2).

### 2.2. Preparation of MJGT_EE

MJGT (200 g) was subjected to heat reflux extraction in 4 L of 70% ethanol for 60 min at 70 °C followed by vacuum concentration for ethanol recovery; then, the ethanol extract obtained was dried and stored at 4 °C until use.

### 2.3. Animal Grouping, Construction of the IBS-C Model, and Sample Collection

Six pregnant specific-pathogen-free (SPF) rats were purchased from Si Pei Fu Biotechnology Co., Ltd. (Beijing, China). The rats were provided clean water, fed daily, and reared under a 12-h light–dark cycle. At 7 days of age, 24 offspring rats were randomly selected and subjected to 3 h of maternal separation from 9 am to 12 noon daily for 14 days. Subsequently, the rats were assigned to three groups: the model (MG), positive drug (MSP) (1 mg/kg of mosapride), and 70% MJGT ethanol extract (MJGT_EE) (17 mg/mL, determined based on the daily dose for humans, with 0.1% dimethyl sulfoxide added in each group for solubilization) groups. Eight rats that were not subjected to maternal separation were assigned to the negative control (NC) group (saline). With the exception of animals in the NC group, animals in all the other groups were administered ice water at 0–4 °C (1.5 mL/rat) via gavage for 14 days. A fecal water content (FWC) measurement and intestinal sensitivity testing were performed to confirm successful IBS-C modeling. Subsequently, drug administration was performed in the different groups for 30 days via gavage.

Next, rats were selected from each group and sacrificed. Then, two 5 × 5-mm proximal colonic tissue specimens were collected from each sacrificed rat and washed thrice with saline for intestinal content removal. One specimen was fixed in 4% paraformaldehyde for immunohistochemical analysis, and the other was stored at −80 °C for western blot analysis. In addition, the cecal contents of the sacrificed rats were collected in 1 mL sterile centrifuge tubes and immediately stored at −80 °C. All specimens were transported on dry ice prior to testing.

All animal experiments were approved by the laboratory animal ethics committee of Shanxi Agricultural University (Taigu, China) (Approval No.: SXAU-EAW-2018R.0406001) and performed in accordance with the regulations and guidance of this committee.

### 2.4. Measurement of FWC

On days 14 and 28 of the IBS-C model construction and drug administration periods, respectively, the rats were separated with each rat reared individually, and the amount of feces passed out within 24 h by the rats in each group was recorded for FWC calculation. Then, the wet weight of the feces was measured using an electronic balance; next, the feces was dried at 105 °C to a constant weight and the dry weight of the feces was recorded. FWC was calculated using Equation (1).

water content% = [wet weight of feces (g) − dried weight of feces (g)]/wet weight of feces (g) × 100% (1)

### 2.5. Measurement of Intestinal Sensitivity

The smallest threshold colorectal distention (CRD) volume was also measured on days 14 and 28 of the IBS-C model construction and drug administration periods, respectively. Each rat was anesthetized using a small amount of diethyl ether and placed in a rat holder. Then, a glycerol-lubricated urinary catheter with an attached balloon was inserted into the colorectum of each rat and taped to the base of the tail, with the end of the balloon positioned approximately 1 cm away from the anus. When the rats regained consciousness and were fully acclimatized to the environment for 30 min, normal saline at 26–28 °C was injected into the balloon, and the smallest injection volume that induced CRD in the rats was recorded. This process was repeated thrice at 15-min intervals, and the smallest threshold volume was calculated by taking the average of the three volumes.

### 2.6. Effects of MJGT_EE on Gastric Emptying and Small Intestinal Propulsion

After continuous gavage for 29 days, the rats were starved for 24 h but allowed access to water. A semi-solid paste was prepared following a slightly modified version of the method described by [44]; first, 10 g of sodium carboxymethyl cellulose was dissolved in 250 mL of distilled water. Then, 16 g of milk powder, 8 g of glucose, 8 g of starch, and 2 g of activated charcoal powder were added to this solution, and the resulting mixture was uniformly mixed to obtain 300 mL of a black semi-solid paste. This paste was stored in a refrigerator and warmed to a temperature of 20 °C before use. At the end of the drug administration period (day 30), 2 mL of the semi-solid paste was measured (Mp: mass of paste) and administered to each rat by gavage. Forty minutes following administration, the animals were anesthetized using pentobarbital sodium (40 mg/kg) and sacrificed. Then, the total mass of the stomach (Mfs), the net mass of the stomach (Mns), the total distance from the pylorus to the ileocecal junction (Lt), and the distance from the pylorus to the front edge of the black semi-solid paste (Lc) were measured. The gastric emptying and small intestinal propulsion rates were calculated using Equations (2) and (3), respectively, as represented below:

Gastric emptying rate (%) = [1 − (Mfs − Mns)/Mp] × 100%(2)

Small intestinal propulsion rate (%) = (Lc/Lt) × 100%(3)

### 2.7. Hematoxylin and Eosin (H&E) Staining and Immunohistochemistry

For H&E staining, colonic tissues were fixed in 10% neutral buffered formalin solution, sectioned, deparaffinized at 65 °C, rehydrated, and stained using an H&E staining kit (Solarbio, G1121).

For immunohistochemical analyses, colonic tissue specimens were sectioned, deparaffinized, and rehydrated. High-temperature antigen retrieval was performed on the tissue specimens using trisodium citrate, and endogenous peroxidase activity and non-specific binding sites in the specimens were blocked with H_2_O_2_ and goat serum, respectively (Zhongshan Jinqiao ZLI-9022). Anti-5-HT antibodies (primary antibodies; 1:1000) were added to the tissue sections and incubated at 4 °C for 12 h. Next, goat anti-rabbit IgGs (secondary antibodies; 1:100) and the peroxidase-antiperoxidase complex (PAP; 1:100) were successively added to the samples and incubated for 1 h at 37 °C each time; staining was performed using diaminobenzidine (DAB)/H_2_O_2_. The sections were adequately rinsed with 0.01 mol/L PBS (Na_2_HPO_4_ 8 mM, NaCl 136 mM, KH_2_PO_4_ 2 mM, KCL 2.6 mM, pH: 7.4) in between the steps described above. After staining, the sections were mounted onto gel-coated slides, dehydrated using graded alcohol, cleared in xylene, mounted, and observed under an optical microscope (Olympus BX-51 biological microscope, white light, Japan, Olympus). Five high-power (200×) fields of view were randomly selected for the calculation of the average optical density (AOD, IOD/Area) using Image-Pro Plus 6.0 (Media Cybernetics, Silver Spring, MD, USA).

### 2.8. Western Blot Analysis

For each rat, 50 mg of colonic tissue was precisely weighed and placed in an Eppendorf tube. First, an appropriate amount of protease inhibitor-containing cell lysis buffer was added to the tissue and the mixture was homogenized using an electric homogenizer. Next, the sample loading buffer was added to the homogenate, and the mixture was boiled for 5 min; then, the resulting mixture was subjected to sodium dodecyl sulfate polyacrylamide gel electrophoresis (SDS-PAGE). Separated proteins were transferred onto a polyvinylidene fluoride (PVDF) membrane and blocked with 5% skimmed milk powder. Second, primary antibodies against 5-hydroxytryptamine receptor 3 (5-HT_3_R; bs-2126R), 5-hydroxytryptamine 4 receptor (5-HT_4_R; bs-12054R), the serotonin transporter (SERT, bs-1893R), tryptophan hydroxylase 1 (TPH1; bs-1215R), tryptophan hydroxylase 2 (TPH2; bs-8729R), calmodulin (CaM; bs-3666R), or myosin light chain kinase (MLCK) (Bioss Biotechnology Co., Ltd., Beijing, China) were added (1:1000) to the membrane and incubated at 4 °C for 12 h. Next, the membrane was washed thrice with TBST buffer (T1081, Solarbio Science & Technology Co., Ltd., Beijing, China); then, secondary antibodies (HRP-conjugated Affinipure Goat Anti-Mouse IgG (H+L) or HRP-conjugated Affinipure Goat Anti-Rabbit IgGs (H+L); 1:5000) were added to the membrane and incubated at room temperature for 1 h. Finally, at the end of the incubation, the membrane was again washed thrice with the TBST buffer, developed, exposed in a dark room, and imaged using a ChemiDoc MP Imaging System (Bio-Rad Laboratories Inc., Hercules, CA, USA). GAPDH or α-tubulin was used as the housekeeping protein.

### 2.9. 16S rDNA Sequencing

DNA extraction: Total microbiota DNA was extracted from rat fecal samples using the E.Z.N.A.^®^ soil DNA kit (Omega Bio-Tek, Norcross, GA, USA) following the instructions of the manufacturer. The quality of the extracted DNA was evaluated via 1% agarose gel electrophoresis, and DNA concentration and purity were measured using the NanoDrop2000 (Thermo Fisher Scientific, Waltham, MA, USA) device.

16S rRNA gene amplification and sequencing through the polymerase chain reaction (PCR): The V3–V4 variable regions of the 16S rRNA gene were subjected to PCR (ABI GeneAmp^®^ 9700, ABI, Los Angeles, CA, USA) using the 338F (5′-ACTCCTACGGGAGGCAGCAG-3′) and 806R (5′GGACTACHVGGGTWTCTAAT-3′) primer sequences under the following cycling conditions: initial denaturation at 95 °C for 3 min, 27 cycles of denaturation at 95 °C for 30 s, annealing at 55 °C for 30 s, extension at 72 °C for 30 s, stable extension at 72 °C for 10 min, and storage at 4 °C. The PCR reaction system was constituted of 4 μL of 5×TransStart FastPfu buffer, 2 μL of 2.5 mM dNTPs, 0.8 μL of forward primer (5 μM), 0.8 μL of reverse primer (5 μM), 0.4 μL of TransStart FastPfu DNA polymerase, 10 ng of template DNA, and ddH_2_O to make up a final volume of 20 μL. PCR was performed in triplicate for each sample.

Illumina Miseq sequencing: DNA fragments were recovered from a 2% agarose gel after mixing, purified using the AxyPrep DNA Gel Extraction Kit (Axygen, San Francisco, CA, USA), detected using 2% agarose gel electrophoresis, and quantified using a Quantus™ Fluorometer (Promega, Madison, WI, USA). Sequencing libraries were constructed using the NEXTFLEX Rapid DNA-Seq Kit (Bioo Scientific, Austin, TX, USA) according to the following steps: (1) adaptor ligation; (2) magnetic bead screening for the removal of self-ligated adaptors; (3) library template enrichment via PCR amplification; (4) magnetic bead recovery of PCR products to obtain the final sequencing libraries. Sequencing was ultimately performed on the Illumina MiSeq PE300 platform (Majorbio Bio-Pharm Technology Co., Ltd., Shanghai, China), and raw sequencing data were uploaded to the NCBI Sequence Read Archive (SRA) (Accession No.: PRJNA906308).

Data processing: Reads were filtered by removing bases with tail quality values of less than 20 using a 50-bp window. If the average quality value within the window was less than 20, bases were trimmed from the back end starting from the window. Reads with lengths < 50 bp after quality control were filtered and those containing N bases were removed. Based on the extent of overlap between the paired-end reads, read pairs were merged into a sequence with a minimum overlap length of 10 bp. Sequences that exceeded the maximum overlap region mismatch ratio allowed (0.2) were removed. Samples were distinguished based on the barcodes at the head and tail ends of sequences and primers, and sequence directions were adjusted based on the number of permitted mismatches (allowable barcode mismatches: 0; maximum allowable primer mismatches: 2). Using the UPARSE software (http://drive5.com/uparse/, version 7.1, accessed on 20 June 2021) package, operational taxonomic unit (OTU) clustering was performed based on a 97% similarity threshold, and chimeric sequences were removed. Each sequence was subjected to species classification and annotation using the RDP classifier and compared against the SILVA 16S rRNA database (version 138), with the confidence threshold set at 70%.

### 2.10. Identification of the Four Chemical Component Types in MJGT_EE with HPLC

Chromatographic conditions: Chromatographic system: Agilent Technologies; chromatographic column: Agilent 5 TC-C18(2), 250 mm × 4.6 mm, 5 μm; flow rate: 1 mL/min; column temperature: 25 °C; injection volume: 10 μL; mobile phase: 0.3% acetic acid (A)-methanol (B) (0–22 min: 32% B, 22–23 min: 32–37% B, 23–36 min: 37% B, 36–37 min: 37–45% B, 37–46 min: 45% B, 46–47 min: 45–60% B, 47–60 min: 60–80% B); UV absorption of eriodictyol and eriodicty-7-O-glucoside was monitored at 284 nm, while monitoring of luteolin and luteolin-7-O-glucoside was at 350 nm..

Preparation of mixed control solution (S1): A mixed solution containing eriodictyol-7-O-glucoside (0.348 mg/mL), eriodictyol (0.200 mg/mL), luteolin-7-O glucoside (0.270 mg/mL), and luteolin (0.156 mg/mL) in methanol was prepared as the control.

Preparation of test solution (S2): Dried MJGT was pulverized and passed through a 40-mesh sieve. A total of 1.7 g of MJGT was precisely weighed and placed in a 250-mL distillation flask. Then, 70% ethanol was added to the flask, which was securely stoppered and weighed. The contents of the flask were subjected to heat reflux extraction for 60 min and cooled to room temperature. Then, 70% ethanol was added to the flask to make up for the lost weight, and the contents of the flask were filtered through a 0.22-μm organic filtration membrane to obtain the test solution.

### 2.11. Statistical Analysis

All data are expressed as the mean ± standard error of the mean (SEM). SPSS version 26.0 (SPSS, Inc., Chicago, IL, USA) was used for statistical comparison of data via one-way analysis of variance (ANOVA). Differences were considered statistically significant when *p* was less than 0.05. Lowercase and uppercase letters were used to denote the results of comparisons at significance levels of 0.05 and 0.01, respectively. All graphs were plotted using GraphPad Prism version 7.0 (GraphPad software, Inc., La Jolla, CA, USA).

## 3. Results

### 3.1. Evaluation of the IBS-C Model

**Figure 1:**
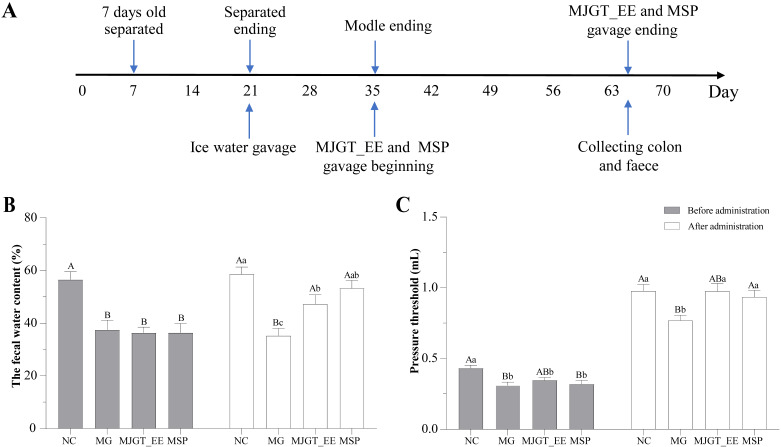
The timeline from rat model establishment, administration to sampling (**A**). Comparison of fecal water content (**B**) and pressure threshold (**C**) in rats of different groups. Capital and lowercase letters above the bar indicate the difference significance at the 0.01 or 0.05 levels, respectively. (*n* = 8 for each group). NC: negative control group; MG: model group, MSP: mosapride group, MJGT_EE: Mao Jian Green Tea ethanol extract group.

Figure 1A shows the process flow diagram for model construction and administration. There was a significant decrease in FWC in the MG group (before administration; *p* < 0.01; Figure 1B). However, rats administered MJGT_EE or MSP exhibited a significant recovery in FWC, with the recovered FWC in the MSP group being similar to that in the NC group (after administration).

Visceral sensitivity changes constitute some of the most important pathophysiological characteristics of IBS patients. In this study, the smallest threshold CRD volume was adopted as a measure of visceral sensitivity in the different groups. A significant decrease in the smallest threshold CRD volume in the MG group was observed (before administration; *p* < 0.05; Figure 1C), indicating a significant increase in rat visceral sensitivity. After drug administration, the smallest threshold CRD volume was restored to normal levels in the MJGT_EE and MSP groups as compared to the MG group (after administration; Figure 1C).

### 3.2. Effects of MJGT_EE on Gastric Emptying and Small Intestinal Propulsion in IBS-C Rats

**Figure 2:**
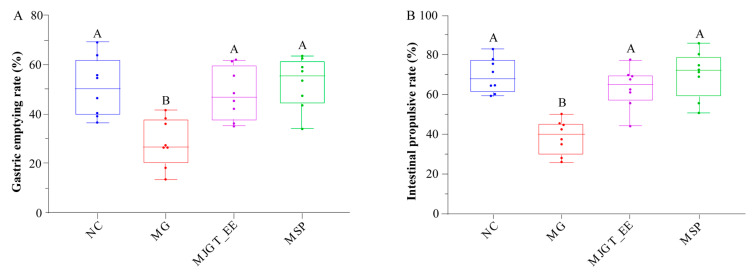
Effect of MJGT_EE on gastric emptying rate (**A**) and intestinal propulsive rate (**B**) in vivo. Capital letters above the bar indicate the difference significance at the 0.01 level. (*n* = 8 for each group). NC: negative control group; MG: model group, MSP: mosapride group, MJGT_EE: Mao Jian Green Tea ethanol extract group.

Previous studies have demonstrated that IBS-C patients exhibit delayed gastric emptying and small intestinal transit [45,46]. Therefore, these two indicators were selected and evaluated in our in vivo experiments. As compared to rats in the NC group, those in the MG group exhibited a significant decrease in gastric emptying and small intestinal propulsion rates (*p* < 0.01). MJGT_EE administration restored both gastric emptying and small intestinal propulsion rates to levels similar to those in the NC group (*p* < 0.01); the treatment effects of MJGT_EE were in line with those of MSP (Figure 2A,B).

### 3.3. Effect of MJGT_EE on the Colonic Tissue Morphology

**Figure 3:**
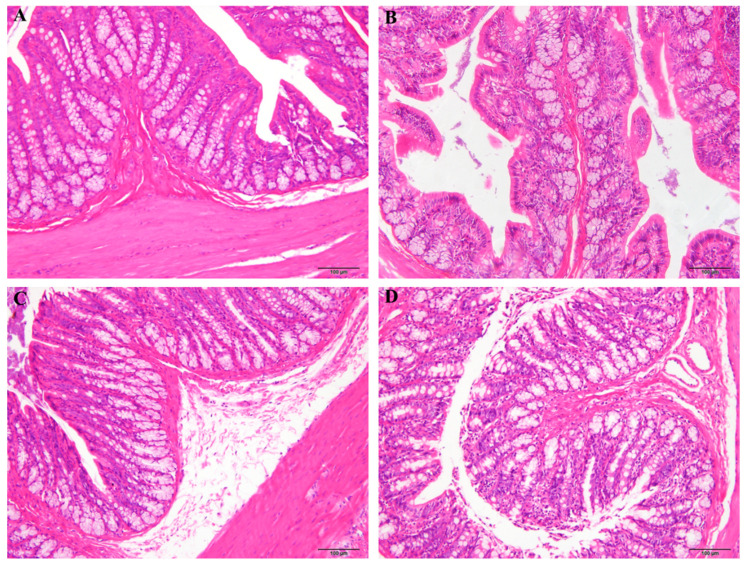
H&E staining to observe the effect of MJGT_EE on the colonic tissues of IBS-C rats (200×). (**A**) The colonic tissues from normal rats; (**B**) the colonic tissues from rats with IBS-C; (**C**) the colonic tissues of IBS-C rats after 30 days of MJGT_EE treatment; (**D**) the colonic tissues of IBS-C rats after 30 days of MSP treatment. (*n* = 8 for each group). NC: negative control group; MG: model group, MSP: mosapride group, MJGT_EE: Mao Jian Green Tea ethanol extract group.

Rat colonic tissues were subjected to H&E staining for morphological evaluation. Clear structures were observed in the various colonic tissue layers. Mucosal epithelial cells exhibited a simple columnar structure, with cells and glands arranged in an orderly manner; in addition, normal colonic crypts were present. No significant inflammatory cell infiltration or pathological damage, interstitial hyperemia or edema, ulcerations, or organic lesions were observed. These findings indicated that the method used for model construction did not induce organic lesion development (Figure 3A,B), and that both the MJGT_EE (Figure 3C) and MSP (Figure 3D) treatments had no effects on rat tissue morphology.

### 3.4. Evaluation of the Accumulation of 5-HT in the Colonic Tissue Impacted by MJGT_EE

**Figure 4:**
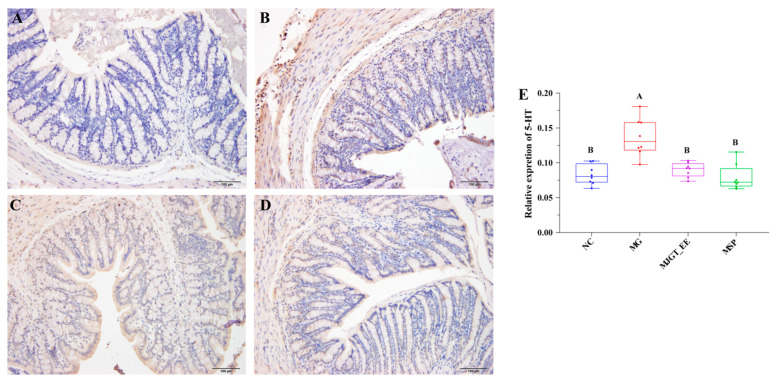
Immunohistochemical staining analysis to observe the effect of MJGT_EE on the colonic tissues of IBS-C rats. (**A**) The colonic tissues from normal rats; (**B**) the colonic tissues from rats with IBS-C; (**C**) the colonic tissues of IBS-C rats after 30 days of MJGT_EE treatment; (**D**) the colonic tissues of IBS-C rats after 30 days of MSP treatment; (**E**) the relative expression of 5-HT. Capital letters above the bar indicate the difference significance at the 0.01 level. (*n* = 8 for each group). NC: negative control group; MG: model group, MSP: mosapride group, MJGT_EE: Mao Jian Green Tea ethanol extract group; 5-HT: 5-hydroxytryptamine.

To determine whether MJGT_EE affects 5-HT expression, 5-HT accumulation in rat colonic tissues was evaluated with the immunohistochemical method. As shown in Figure 4, 5-HT was mainly distributed in the submucosal and muscular layers of the colon. Calculated AOD values indicated that colonic 5-HT secretion significantly increased in the MG group as compared to the NC group (*p* < 0.01; Figure 4A,B), suggesting that the model construction method induced an increase in colonic 5-HT levels. After MJGT_EE or MSP intervention, colonic 5-HT secretion significantly decreased in these groups as compared to the MG group (*p* < 0.01, Figure 4C,D). Therefore, MJGT_EE and MSP exhibited similar regulatory effects on 5-HT, and this is consistent with the results described above (Figure 4E).

### 3.5. Evaluation of the Expression of TPH1 and TPH2 in the Colonic Tissue Impacted by MJGT_EE

**Figure 5:**
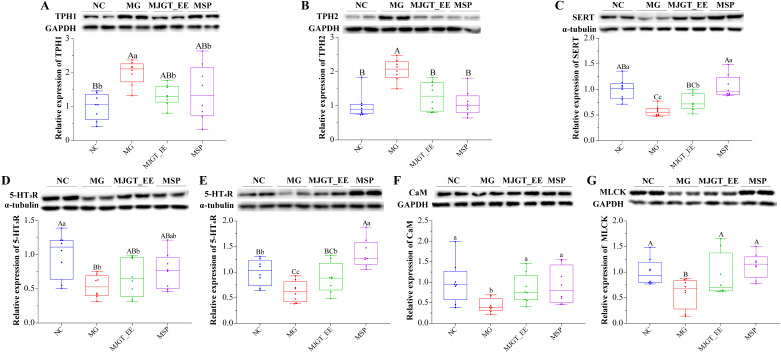
Western blotting analysis of TPH1 (**A**), TPH2 (**B**), SERT (**C**), 5-HT_3_ receptors (**D**), 5-HT_4_ receptors (**E**), CaM (**F**), and MLCK (**G**) in colonic tissues of each group. Capital and lowercase letters above the bar indicate the difference significance at the 0.01 or 0.05 levels, respectively. (*n* = 8 for each group). NC: negative control group; MG: model group, MSP: mosapride group, MJGT_EE: Mao Jian Green Tea ethanol extract group; 5-HT: 5-hydroxytryptamine; TPH1: tryptophan hydroxylase 1; TPH2: tryptophan hydroxylase 2; SERT: serotonin transporter; CAM: calmodulin; MLCK: myosin light chain kinase; 5-HT_3_R: 5-HT_3_ receptor; 5-HT_4_R: 5-HT_4_ receptor.

To further evaluate the cause of the decrease in 5-HT synthesis observed, western blotting was performed to quantitatively determine changes in the levels of tryptophan hydroxylases (TPHs), which are key enzymes of the 5-HT metabolic pathway. A significant increase in the expression levels of TPH1 and TPH2 in the colons of IBS-C rats was identified (MG group) (*p* < 0.01), suggesting that changes in 5-HT expression levels in the model were significantly associated with TPH synthesis (Figure 5A,B). Following treatment with MJGT_EE, the expression levels of both TPH1 and TPH2 decreased (*p* < 0.05, Figure 5A,B), and its effects were similar to those of the positive drug, MSP. Treatment with both MSP and MJGT_EE restored TPH expression to levels similar to those of the NC group (Figure 5A,B).

### 3.6. Effect of MJGT_EE on SERT Expression in the Colon

5-HT synthesized in the intestines can be transported by the SERT from the interstitial spaces of the lamina propria to the mucosal epithelial cells and presynaptic neurons. This process is known as 5-HT reuptake [47]. Thus, the SERT content is also a major factor that affects 5-HT expression. IBS-C negatively affected colonic SERT expression in rats in the MG group as compared to rats in the NC group (*p* < 0.01). Following drug administration (MJGT_EE and MSP), SERT expression levels were restored to levels similar to those observed in rats in the NC group at the 0.01 level (Figure 5C).

### 3.7. Effect of MJGT_EE on the Expression of 5-HT_3_R and 5-HT_4_R in the Colon

Among the seven confirmed 5-HT receptor subtypes, 5-HT_3_R and 5-HT_4_R are significantly related to gastrointestinal motility and pain sensitivity. Therefore, these two indicators were evaluated to determine the effects of the drug. As compared to rats in the NC group, those in the MG group exhibited a significant decrease in 5-HT_3_R and 5-HT_4_R expression (*p* < 0.01). Both MSP and MJGT_EE restored 5-HT_4_R expression levels (*p* < 0.05, Figure 5D,E), with the expression levels in rats in the MSP group being higher than those in rats in the NC group (*p* < 0.01). However, a similar trend was not observed with 5-HT_3_R expression.

### 3.8. Effect of MJGT_EE on CaM-MLCK Signaling Pathway

The CaM-MLCK pathway is a classical downstream pathway of 5-HT_4_R that regulates smooth muscle contraction. As compared to rats in the NC group, those in the MG group exhibited significantly lower CaM (*p* < 0.05) and MLCK (*p* < 0.01) expression levels. Following treatment with MJGT_EE and MSP, both CaM and MLCK expression levels were restored to levels similar to those in rats in the NC group (Figure 5F,G).

### 3.9. Effects of MJGT_EE on IBS-C Rat Gut Microbiota

**Figure 6:**
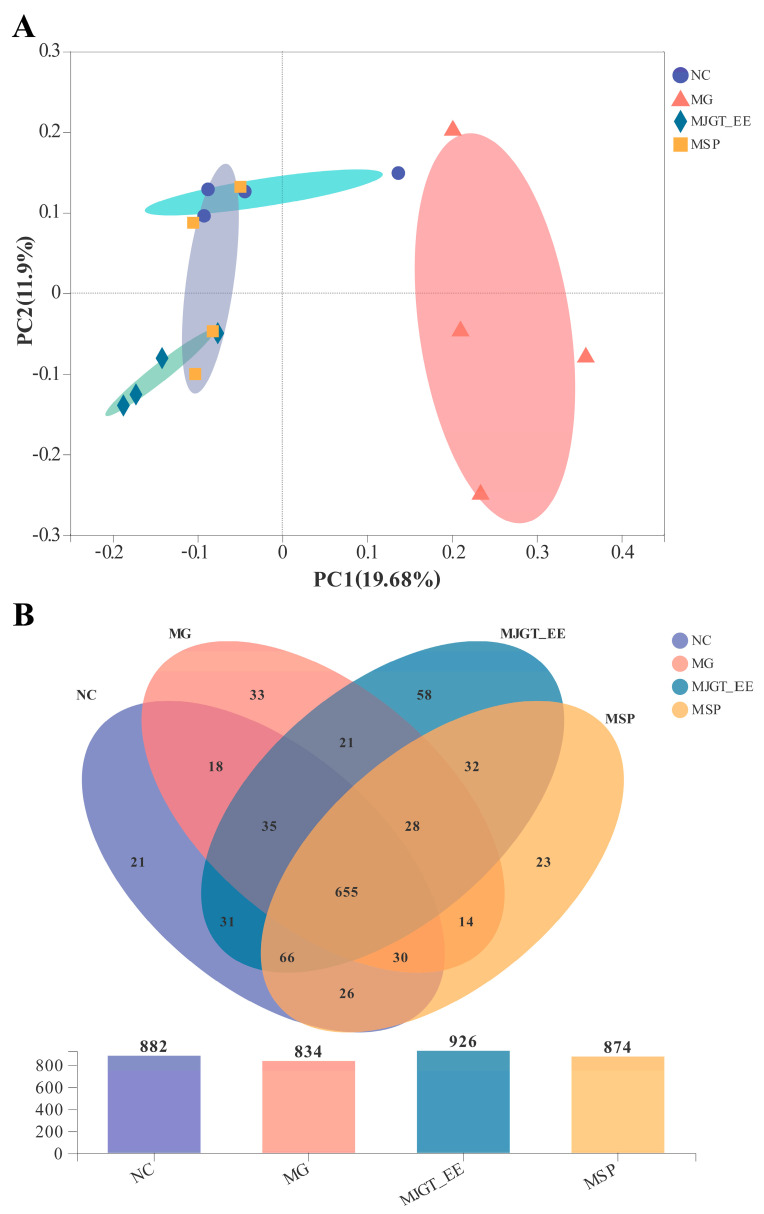
PCoA analysis of the OTU level of intestinal flora in different groups rats (**A**); venn diagram of the distribution of OTUs in the four groups (**B**). (*n* = 4 for each group). NC: negative control group; MG: model group, MSP: mosapride group, MJGT_EE: Mao Jian Green Tea ethanol extract group.

IBS-related pathophysiological changes involve alterations in gut microbiota composition or microbiota dysbiosis [48,49,50]. Therefore, 16S rDNAs of the gut microbiota in rats in the different treatment groups were analyzed. β diversity analysis revealed the existence of significant differences in the microbiota between rats in the MG and NC groups (Figure 6A). The number of OTUs decreased from 882 (NC) to 834 (MG) following model construction but increased following drug administration (MJGT_EE and MSP). Notably, the number of OTUs was highest following treatment with MJGT_EE (926) (Figure 6B). The ACE, Chao, Sobs, and Shannon α diversity indices also showed that the diversity of the gut microbiota in rats with IBS-C was lower than that in rats in the NC group; however, this diversity was restored following treatment with MJGT_EE or MSP (*p* > 0.05) (Figure S1).

**Figure 7:**
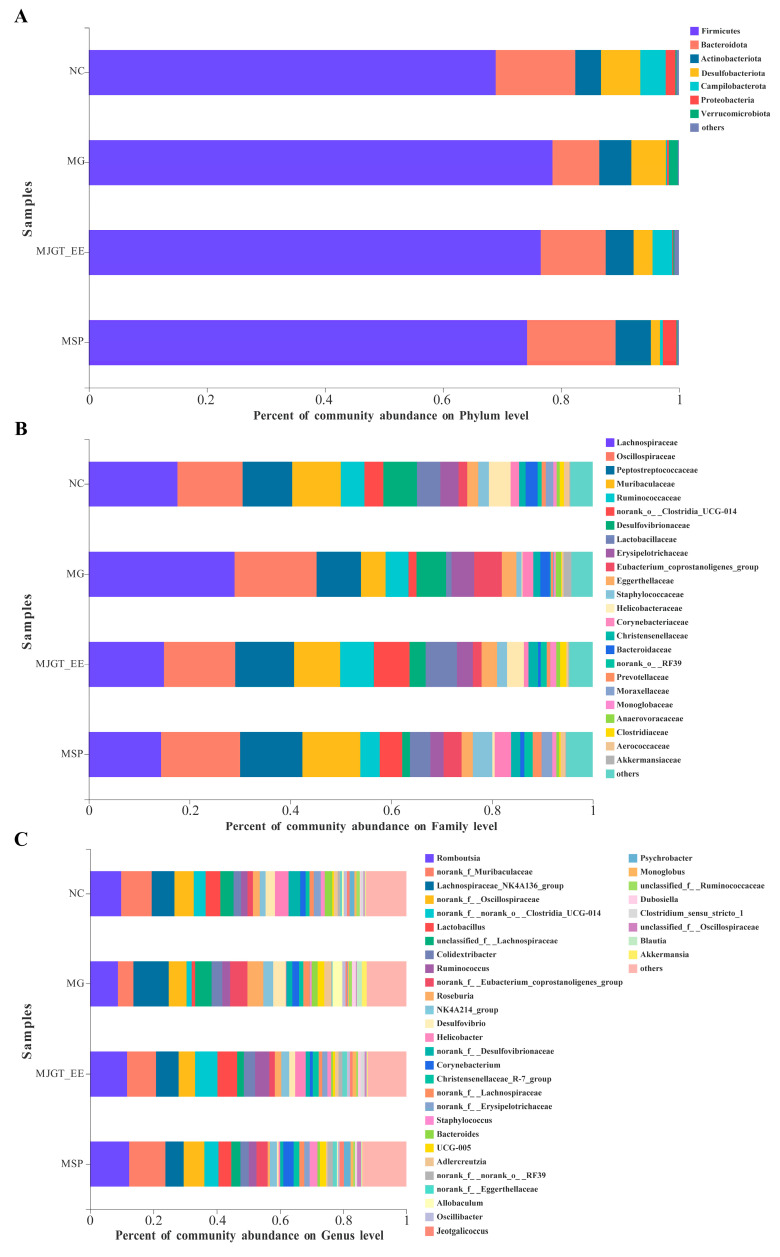
The variations in intestinal flora composition are displayed the phylum (**A**) and family (**B**) and genus level (**C**), respectively. (*n* = 4 for each group). NC: negative control group; MG: model group, MSP: mosapride group, MJGT_EE: Mao Jian Green Tea ethanol extract group.

At the phylum level, bacteria of the phyla Firmicutes and Bacteroidota accounted for the greatest proportion of microbes, with their proportions in the NC, MG, MJGT_EE, and MSP groups being 68.95% and 13.49%, 78.57% and 7.94%, 76.59% and 11.00%, and 74.27% and 15.00%, respectively (Figure 7A). The Bacteroidetes/Firmicutes ratio in the MG group (0.10) decreased onefold as compared to that in the NC group (0.20). This is consistent with the previously reported significant increase in Firmicutes bacterial strain counts [51] and the decrease in the Bacteroidetes/Firmicutes ratio in IBS-C patients [52]. Following drug administration, the Bacteroidetes/Firmicutes ratio increased in the MJGT_EE group (0.14) but was still lower than that in the MSP group (0.20).

At the family level, changes in the abundance of bacteria of the Prevotellaceae family in the different groups (0.80% and 0.43% in the NG and MG groups, respectively) followed a similar trend to those of bacteria of the Bacteroidota phylum, i.e., the restoration of bacterial abundance was more significant in the MSP group (1.75%) than in the MJGT_EE group (0.69%). The proportion of Clostridia_UCG-014 strains in the MG group (1.59%) decreased by 58.6% as compared to that in the NC group (3.75%); following drug administration, in the MJGT_EE group, this proportion (7.08%) was 4.45 and 2.79 times that in the MG and MSP groups, respectively. Linear discriminant effect size analysis (LFEse) revealed Clostridia_UCG-014 to be the only characteristic bacterium enriched in the MJGT_EE group (LDA > 4, Figure S2). There was an increase in the abundance of some bacterial families in IBS-C rats. For instance, the abundance of bacteria of the Lachnospiraceae family in the MG group (28.94%) was 1.64 times that in the NC group (17.61%); however, this increase was reversed following treatment with MJGT_EE (14.94%) and MSP (14.37%). The abundance of bacteria of the Corynebacteriaceae family ranged from 1.68% in the NC group to 2.16% in the MG group, but decreased to 0.92% following treatment with MJGT_EE. The proportions of some bacteria remained unchanged before and after model construction, but changed following drug treatment. For instance, bacteria of the Ruminococcaceae family accounted for 4.54% and 4.68% of the microbiota in rats in the NC and MG groups, respectively. Their abundance increased to 6.67% following treatment with MJGT_EE but decreased to 3.86% following treatment with MSP (Figure 7B).

At the genus level, the proportion of *Lactobacillus* sp. decreased by approximately 76.08% in the MG group (1.11%) as compared to the NC group (4.64%). Their abundance was restored following the administration of MJGT_EE (6.19%) and MSP (4.04%), with MJGT_EE inducing a 0.33-fold increase in their abundance as compared to that in the NC group (Figure 7C).

### 3.10. Identification of the Four Chemical Component Types in MJGT_EE

MJGT_EE was analyzed using high-performance liquid chromatography (HPLC). Mixed control (S1) and test (S2) solutions were prepared as described in Section 2.10 and analyzed under appropriate chromatographic conditions. Through a comparative analysis of retention time and ultraviolet (UV) spectra, four main chromatographic peaks, which represented luteolin-7-O-glucoside (Figure S3(1)), luteolin (Figure S3(2)), eriodictyol-7-O-glucoside (Figure S3(3)), and eriodictyol (Figure S3(4)), were identified in MJGT_EE.

## 4. Discussion

In IBS patients, gastrointestinal motility disorders are often accompanied by visceral hypersensitivity responses, which are exaggerated sensational responses to environmental stimuli, possibly induced by alterations in the processing of afferent signals from visceral neurons [53]. The IBS-C rat model can be constructed via water limitation, ice water stimulation, and the maternal separation plus ice water stimulation. The first two methods ignore the psychological factors in the pathogenesis of IBS-C, so the last one that is more closely related to the patient’s pathogenesis was chosen in the study. The chronic stress in newborn rats was applicated using maternal separation to induce stable changes in the central nervous system through the hypothalamic–pituitary–adrenal axis (HPA), as well as cognitive and emotional functions. This led to the gradual development of a disease state characterized by increased visceral sensitivity at the level of the large intestines after the maturation of the animals. Subsequently, to establish the IBS-C model, ice water at 4 °C was administered daily via gavage to the rats to induce symptoms of constipation. A comparison of the FWC and smallest threshold CRD volume between the MG and drug administration groups showed that drug administration not only reversed the significant decrease in FWC, but also decreased intestinal sensitivity in rats in the MG group. Therefore, MJGT_EE is able to reverse the decrease in FWC, as well as the increase in intestinal sensitivity, exhibited by IBS-C rats. Another characteristic symptom of IBS is the chronic disruption of normal gastrointestinal peristaltic activity [54], which mainly manifests as delayed gastric emptying and small intestinal transport [55]. Our experimental results showed that rats in the MG group exhibited delayed gastric emptying and decreased small intestinal propulsion functions, and these were significantly improved via treatment with MJGT_EE. This provides preliminary evidence of the effects of MJGT_EE in promoting gastrointestinal motility in IBS-C patients. Notably, this method used to construct IBS-C in the present study can only represent one of the psychological factors that cause the onset of IBS.

Studies on the gastrointestinal motility-promoting mechanism of MJGT_EE have shown that it elicits this effect by inhibiting the secretion of 5-HT, which is a typical indicator of alterations in IBS patients. In addition, 5-HT plays a key role in the regulation of gastrointestinal motility, secretion, and sensation [56,57]. In this study, colonic tissue H&E staining revealed no inflammatory exudates nor pathomorphological changes. This is consistent with the findings of previous studies, which reported that IBS patients typically do not manifest organic lesions [58] and that IBS-C rarely induces inflammatory responses. To further evaluate the causes of alterations in the 5-HT signaling system, we investigated rate-limiting enzymes that directly affect 5-HT synthesis, i.e., TPH (TPH1 and TPH2) [59,60], as tryptophan (Trp) is a precursor of 5-HT [61] that is first converted to 5-hydroxytryptophan (5-HTP) under the action of TPH and is subsequently converted to 5-HT under the action of 5-HTP decarboxylase (5-HTPDC). TPH plays a vital role in the conversion of Trp to 5-HTP [62,63] and directly affects 5-HT secretion. Synthesized 5-HT, which is stored in enterochromaffin (EC) cells, is released into the lamina propria in response to luminal pressure, as well as chemical or mechanical stimuli, where it interacts with nerve endings and immune cells [64]. As with 5-HT release, its deactivation is of equal importance in maintaining dynamic equilibrium. The SERT plays an indispensable role in 5-HT deactivation as it transports 5-HT from the interstitial spaces of the lamina propria to the intestinal mucosal cells and presynaptic neurons and is subsequently involved in 5-HT degradation. Insufficient SERT synthesis leads to 5-HT accumulation [65]. This induces high contractility in the digestive tract smooth muscles, as well as high gland sensitivity and increased endocrine secretion, which result in diarrhea and pain. A significant increase in 5-HT levels in the MG group was observed as compared to the NC groups, and this was the possible cause of the abnormalities in gastrointestinal motility; however, treatment with MJGT_EE exerted a significant downregulatory effect on 5-HT levels. These findings are consistent with the trend observed in the changes in TPH expression in the different groups, demonstrating that MJGT_EE reduced 5-HT secretion by decreasing TPH synthesis. The decrease in SERT expression was identified in the MG group; however, this expression was upregulated following treatment with MJGT_EE, indicating that drug administration inhibited excessive 5-HT accumulation. Therefore, MJGT_EE evidently restores abnormally increased 5-HT levels by regulating TPH and SERT expression.

MJGT_EE regulates gastrointestinal responses by regulating 5HT_4_R. 5-HT released from EC cells can regulate gastrointestinal motility by effectively activating 5-HT_3_R and 5-HT_4_R at the vagal afferent nerve endings of the intestinal mucosa. Consequently, the activation of these receptors enhances gastrointestinal transport [66,67]. Our findings indicated that the expression levels of both 5-HT_3_R and 5-HT_4_R were downregulated in IBS-C rats, and that their expression levels were negatively correlated with visceral sensitivity, and this is consistent with findings reported in the literature [68,69]. Interestingly, in a similar way as MSP, a selective 5-HT_4_R agonist, MJGT_EE only exerted restorative effects on 5-HT_4_R expression but did not affect 5-HT_3_R expression. This phenomenon could be explained by the fact that 5-HT released by EC cells can mediate digestive functions through the activation of endogenous or exogenous sensory nerve endings at high concentrations [70,71] and activate 5-HT_4_ or 5-HT_1_P receptors at a low concentrations, thereby regulating gastrointestinal motility [72,73]. MJGT_EE reduced 5-HT synthesis, inducing lower 5-HT luminal concentrations in the colon; this may explain the lack of effect of MJGT_EE on 5-HT_3_R secretion. The CaM-MLCK pathway is a core pathway through which 5-HT_4_R regulates downstream smooth muscle contraction. Following the activation of Ca^2+^ ion channels, free Ca^2+^ concentrations within cells rapidly increase, leading to the formation of Ca^2+^–CaM complexes. Consequently, MLCK is activated and induces the phosphorylation of the 19th serine residue on myosin light chain 20 (MLc20). This in turn activates myosin ATPase, which hydrolyzes ATP and coverts the chemical energy it contains to mechanical energy that enables myosin to slide past actin filaments and achieve smooth muscle contraction, ultimately resulting in the acceleration of intestinal peristalsis [74]. The findings of our study also indicate that changes in CaM and MLCK expression in the MG group before and after drug administration were positively correlated with 5-HT_4_R expression, further corroborating the effects of MJGT_EE.

Intestinal microbes would also be involved in the gastrointestinal motility impacted by MJGT_EE. Several clinical studies indicated the variation of gut microbiota composition in IBS patients [75,76,77], and this might be a possible etiology for the disorder. In this study, we found that treatment with MJGT_EE led to the following.

(1) Increase in gut microbiota diversity. The gut microbiota in IBS patients is significantly different from that in healthy individuals and is characterized by lower bacterial diversity [75,78]. We found that the number of OTUs in rats with IBS-C was 5.4% lower than that in normal rats (NC group), however, increased by 11% and 4.8% following treatment with MJGT_EE and MSP, respectively. This indicates that not only was MJGT_EE beneficial in increasing microbiota diversity, but it also elicited effects superior to those of MSP.

(2) Increase in the number of bacteria with known benefit. MJGT_EE increased the counts of some beneficial bacteria such as those belonging to the *Lactobacillus* genus and the Prevotellaceae and Ruminococcaceae families. *Lactobacillus* sp. strains alleviate gastrointestinal diseases [79,80], reduce allergic symptoms [81], and are considered as potential antimicrobial probiotic strains against human pathogens [3,82] through various mechanisms. Bacteria of the Ruminococcaceae family can generate short-chain fatty acids (SCFA) [83], which are generally believed to elicit beneficial effects in the human body, such as improving intestinal health and protecting the intestinal mucosal barrier [84]. This increase in beneficial bacterial counts may be related to the MJGT_EE-induced decrease in 5-HT secretion, as 5-HT directly inhibits the growth of beneficial bacteria [52].

(3) Regulation of gut microbes involved in 5-HT synthesis. Studies have shown that *Corynebacterium* sp. strains promote 5-HT synthesis in tissues [85,86]; we observed an increase in the proportion of *Corynebacterium* sp. strains in rats in the MG group as compared with that in the rats in the NC group; in the MG group, this proportion decreased to approximately half the initial level following treatment with MJGT_EE. The proportions of bacteria of the Lachnospiraceae family, which induce 5-HT biosynthesis and release by EC cells [87], were also restored to levels similar to those in rats in the NC group following treatment with MJGT_EE and MSP. Notably, Clostridia_UCG-014 strains, which are beneficial bacteria associated with tryptophan metabolism and that regulate intestinal homeostasis, were only enriched in rats in the MJGT_EE treatment group [88]. Similar effects were observed for MSP, persuading us to speculate that Clostridia_UCG-014 strains may be key bacterial species that affected gastrointestinal motility in rats in the MJGT_EE group.

Generally, an organism has a core native microbiota that remains relatively stable during adulthood. However, each individual has a unique gut flora profile due to a variety of factors such as gut type, body mass index (BMI) level, frequency of exercise, lifestyle, culture, and diet. Therefore, there is no one best gut microbiota composition [89]. For example, gut microbiota are not only able to respond to the various physiological activities of flavonoids, but also to metabolize them and produce new active products [90]. Through an HPLC analysis, four major MJBT_EE components were identified: eriodictyol-7-O-glucoside, luteolin-7-O-glucoside, eriodictyol, and luteolin. In the previous trial, we found that these four components are the active ingredients in hydro extracts of MJGT that affect gastrointestinal motility [42]. The regulation effect of luteolin and other flavonoids, such as apigenin and quercetin, on muscle tissue contraction in cows were also identified [41]. Thus, we believe that they should also be important components of MJBT_EE to alleviate the symptoms of IBS-C. Notably, whether there are other active ingredients needs to be further investigated.

## 5. Conclusions

MJGT_EE promoted gastrointestinal motility and reduced intestinal sensitivity in IBS-C model rats established via maternal separation. These effects were mainly related to a decrease in 5-HT secretion and an upregulation in 5-HT_4_R expression and were not related to 5-HT_3_R expression. The possible mechanism underlying the effects of MJGT_EE on 5-HT secretion may involve the decrease and increase in TPH and SERT secretion, respectively, while underlying that its effects on 5-HT_4_R expression involve the upregulation of the CaM-MLCK pathway. MJGT_EE also increased microbiota diversity and beneficial bacterial counts, while restoring gut microbiota composition disturbed by IBS-C. Since the flavonoids are important active ingredients in MJGT_EE, the final observations would result from the complicated interactions among flavonoids and gut microbiota bioactivity and a wide range of their metabolites, which are worthy of further investigation.

## Data Availability

Data is contained within the article or Supplementary Material.

## Supplementary Materials

The following supporting information can be downloaded at: https://www.mdpi.com/article/10.3390/foods12051101/s1, Figure S1: Changes of intestinal flora diversity in rats of MJGT_EE group. (*n* = 4 for each group). Figure S2: Linear discriminant effect size (LEfSe) analyses comparing differentially abundant taxa in each group (A); the LDA effect size taxonomic cladogram comparing four groups. Different colors indicate species with significant differences between groups, and the logarithmic LDA score is set to 4 (B). (*n* = 4 for each group). Figure S3 The chromatogram of the MJGT_EE. (A) The mixed control solution of luteolin-7-O-glucoside (1) and luteolin (2) detected at 350 nm; (B) The test solution detected at 350 nm; (C) The mixed control solution of eriodictyol-7-O-glucoside (3) and eriodictyol (4) detected at 284 nm; (D) The test solution detected at 284 nm.
